# The association of fetal growth rate and growth in first year of life with childhood overweight: a cohort study

**DOI:** 10.1038/s41366-024-01637-w

**Published:** 2024-09-21

**Authors:** Magnus Leth-Møller, Ulla Kampmann, Susanne Hede, Per G. Ovesen, Adam Hulman, Sine Knorr

**Affiliations:** 1 Department of Clinical Medicine, Aarhus University, Palle Juul-Jensens Boulevard 99, 8200 Aarhus N, Denmark; 2 Department of Obstetrics and Gynaecology, Aarhus University Hospital, Palle Juul-Jensens Boulevard 99, 8200 Aarhus N, Denmark; 3 Steno Diabetes Center Aarhus, Aarhus University Hospital, Palle Juul-Jensens Boulevard 11, 8200 Aarhus N, Denmark; 4 Healthcare Service for families, Aarhus Municipality, Grøndalsvej 2, 8260 Viby J, Denmark; 5 Department of Public Health, Aarhus University, Bartholins Allé 2, 8000 Aarhus C, Denmark

**Keywords:** Epidemiology, Obesity, Obesity, Paediatrics

## Abstract

**Background:**

There is an increasing focus on the first 1000 days from conception to two years of age as a period of importance for future weight. We aimed to describe the interaction between fetal and infant growth and their association with and ability to predict childhood overweight.

**Methods:**

We used routinely collected fetal growth data from Aarhus University Hospital and child growth data from Aarhus Municipality, 2008–2018. The outcome was overweight at age 5–9 years. The fetal growth rates at weeks 28 and 34 were extracted from individual trajectories using mixed models. We identified patterns of infant BMI *Z*-score growth using latent class analysis and estimated odds ratios of overweight at age 5–9 years dependent on fetal and infant growth. Predictive capabilities were assessed by comparing areas under the ROC-curves (AUCROC) of the prediction models.

**Results:**

In 6206 children, we identified three infancy growth patterns: average, accelerated, and decelerated growth. We found 1.09 (95% CI: 1.06–1.12) greater odds of being overweight for every 10 g/week increase in fetal growth rate at week 34. Compared with average growth, accelerated infant growth was associated with 1.52 (95% CI: 1.20–1.90) greater odds of overweight. Combining fetal and infant growth, children with average fetal growth and accelerated infant growth had 1.96 (95% CI: 1.41–2.73) greater odds of overweight. Fast fetal growth with decelerated infant growth was not associated with being overweight (OR: 0.79 (95% CI: 0.63–0.98)), showing that infant growth modified the association between fetal growth and overweight. When fetal growth was added to a prediction model containing known risk factors, the AUCROC remained unchanged but infant growth improved the predictive capability (AUCROC difference: 0.04 (95% CI: 0.03–0.06)).

**Conclusion:**

Fetal and infant growth were independently associated with overweight, but distinct combinations of fetal and infant growth showed marked differences in risk. Infant, but not fetal, growth improved a prediction model containing known confounders.

## Introduction

Overweight and obesity can be passed on from generation to generation. Children of mothers with overweight often become overweight themselves, and the overweight often persists into adulthood [1]. During the last decade, there has therefore been an increased focus on the first 1000 days of life, from conception to two years of age, as a period of great importance for future health. In this period, both low and high birthweight are associated with the later development of overweight [2]. In-utero growth is often expressed with birthweight as a simple measure. However, birthweight is merely the endpoint of growth throughout pregnancy, and the growth pattern in-utero is now receiving increased attention [3–8]. After birth, infant growth is found to be associated with overweight and fat mass in later childhood [5, 9–11], and generally, catch-up, accelerated, or high-stable growth trajectories are associated with an increased risk of overweight [12, 13]. Intra- and extrauterine growth are often treated as two separate entities. However, infant growth is a continuation of fetal growth, but only a few studies have examined them in continuation. Even if fetal and infant growth is associated with the later development of overweight, this does not necessarily translate into a predictive capability of overweight. Nevertheless, many studies confuse association and prediction and conclude on predictive capabilities even if only associations have been investigated [14].

The aim of this study is to describe both the association and predictive value of fetal growth and growth in infancy in relation to the later development of overweight. We hypothesize that fetal growth with subsequent distinct patterns of growth in the first year of life is associated with the later development of childhood overweight and that fetal and infant growth is predictive of future overweight.

## Materials and methods

### Study design and participants

We conducted a longitudinal cohort study using routinely collected data on children born in Aarhus, Denmark, from 2008–2018 (inclusive). The data originated from three sources: Aarhus Municipality Healthcare service, the electronic patient records from Aarhus University Hospital, and the Danish Fetal Medicine Database. The municipal healthcare records contain information from health visitors (specialty nurses) who conduct home visits four to five times during the child’s first year of life (visits at ages 1–5 days, 2–3 weeks, 2–3 months, 6 months (only first-time parents), and 9–10 months) and who conduct consultations at school (at ages approximately 6, 7, 9, and 15 years). At each visit or consultation, weight and length/height were measured. Participation rates are not registered, but approximately 95% participation has been estimated [15]. Information on fetal growth and maternal health during pregnancy was collected from the electronic patient records at Aarhus University Hospital. Data on birthweight and covariates were, if missing, supplemented from the Danish Fetal Medicine Database [16], a national database that collects information from fetal ultrasound scans and is supplemented from the Danish National Birth Registry. To create latent class trajectories of infant growth, we included all children born between 2008 and 2018 with at least three measurements of weight and length in the first year of life to create trajectory groups based on the largest possible population. For the main analysis, we included singleton children born 2008–2014 (eligible for 5 years of follow-up), with at least one estimated fetal weight after gestational week 18, a recorded birthweight, and a recorded weight and height at the age of 5–9 years. The study was approved by the Danish Patient Safety Authority (3-3013-2665/1) and the Danish Data Protection Agency (1-16-02-619-18). Ethical approval and patient consent were waived because of the epidemiological design and the size of the study population, in accordance with the General Data Protection Regulation in Denmark.

### Variables

The main outcome was overweight at age 5–9 years (inclusive), defined as having a BMI *z*-score of one SD or above as per the WHO definition. The sex-specific BMI *z*-score were calculated using the WHO reference [17]. If multiple measurements were available, we used the latest.

#### Exposure

##### Fetal and infant growth

Information on estimated fetal weight, fetal head circumference, abdominal circumference, and femur length, as well as birthweight and abdominal and head circumference at birth was obtained from the Picture Archiving and Communications System (Astraia, Astraia Software GmbH, München, Germany) at Aarhus University Hospital. If the estimated fetal weight was missing it was calculated by the Hadlock III formula [18]. In Denmark, all pregnant women are offered fetal ultrasound scans at gestational weeks 12 and 20. We included all scans after gestational week 18 and throughout pregnancy, including routine scans (week 20) and additional scans performed on any indication. The scans were performed by trained sonographers or medical doctors. Assessment of fetal growth trajectories is described below in the statistical analysis section. Birthweight *z*-scores were calculated using sex-specific references by Marsal et al. [19] and small and large for gestational age was defined as a birthweight below the 10th percentile or above the 90th percentile, respectively.

For infant growth, we calculated sex-specific BMI *z*-scores using the WHO reference material [20]. These were used to identify distinct patterns of growth (see statistical analysis below).

#### Covariates

Maternal age at booking, smoking status (yes or no), ethnicity, diabetes status during pregnancy (type 1, type 2, or diet- or insulin-treated gestational diabetes), gestational age at birth, delivery mode (cesarean section or vaginal delivery), parity (nulli- or multipara), and pre-pregnancy BMI ( < 18, 18–24.9, 25–29.9, ≥ 30) were obtained from the medical records at Aarhus University Hospital. Ethnicity was grouped as Caucasian, Afro-Caribbean, East Asian, South Asian, or other, but were combined to Caucasian and others for the descriptive statistics. We calculated *z*-scores for abdominal circumference, head circumference, and femur length at ultrasound scans performed at week 19–25 using the respective formula by Chitty et al. [21–23]. The duration of exclusive breastfeeding (no breastfeeding, 0–4 months, ≥ 4 months) was obtained from the Municipal Healthcare Service records.

### Statistical analysis

Population characteristics are reported as the means with standard deviations (SD) for continuous variables and as frequencies (%) for categorical variables.

#### Fetal growth

Using mixed models, we assessed trajectories of fetal growth with estimated fetal weight as a function of time (gestational age) modeled with linear, quadratic, and cubic terms for time. We included random effects for the intercept and linear and the quadratic terms. Birthweights were used in continuation of estimated fetal weights and were included in this analysis. Therefore, each individual had at least two measurements: one ultrasound-estimated fetal weight and one birthweight. For model specifications, see supplementary materials. The mixed model approach was chosen to account for the repeated measures of fetuses. The fixed effects represent the population grand mean, and the random effects represent the individual level deviation from the grand mean. We calculated the individual-specific fetal growth trajectories by combining the random effects with the fixed effects. To use the fetal growth rate as an exposure in the following analyses, we calculated individual fetal growth rates at weeks 28 and 34 to investigate both early and late growth, as both time periods have been shown to be important for later growth and weight [4, 5]. Week 34 was used in the main analysis, and the results from week 28 are provided in the supplementary materials data section.

In a secondary analysis, we also used mixed models to assess the trajectories of femur length, and head and abdominal circumference in those children with two or more ultrasound scans, and we extracted growth rates for each measure at week 34 (model specifications in the supplementary material). To evaluate the possible bias introduced by including women with scans performed in addition to routine care, we compared the characteristics of women receiving only routine scans with those women who received additional scans.

#### Infant growth

Infant growth was described using latent class trajectory analysis. Briefly, in latent class analysis, we assume that the population consists of a number of subgroups of individuals based on the level and shape of growth trajectories [24]. The aim is to define groups with high between-group separation and low within-group variation but with parsimony regarding the number of groups. Each child is given a probability of belonging to each of the groups and is assigned to the group with the highest probability of membership. To estimate the number of groups, we evaluated 2-5 group solutions using posterior group membership probabilities (mean probability of membership to the assigned group compared to belonging to an alternative group), relative entropy (a measure of group separation), and visually inspected scatter plots depicting growth patterns [25] and considered meaningful clinical separations. We used latent class growth mixture modeling to identify BMI z-score trajectories as a function of age using restricted cubic splines as the underlying structure (model specifications in the supplementary material).

#### Associations with overweight

The associations between fetal growth and overweight and between infant growth and overweight were evaluated using logistic regression. We created two models of overweight dependent on 1) fetal growth (per 10 g/week difference) and 2) the infant growth group. Models are reported as unadjusted and adjusted for maternal pre-pregnancy BMI, age, parity, smoking status, and duration of breastfeeding. In a single logistic regression model, we estimated the risk of overweight dependent on growth of the abdominal circumference, head circumference, and femur length at week 34. Confounders were identified a priori using directed acyclic graphs (Supplementary Fig. S1). To examine the combined effect of fetal growth and infant growth, we categorized fetal growth by growth rate quartiles rounded to the nearest fifth. At week 28, we defined fetal growth as slow (1st quartile, ≤ 164 g/week), average (2nd and 3rd quartile, 165–189 g/week), or fast (4th quartile, ≥ 190 g/week), and at week 34, we defined fetal growth as slow (1st quartile, ≤ 184 g/week), average (2nd and 3rd quartile 185–219 g/week), or fast (4th quartile, ≥ 220 g/week). We compared logistic regression with and without interactions between fetal and infant growth using the analysis of variance. We stratified groups for the nine possible combinations of fetal and infant growth and reported the odds ratios (OR). Missing values for categorical variables were included in the adjusted analyses as a missing category.

#### Prediction model

To evaluate the predictive value of fetal and infant growth, we created three prediction models to evaluate whether including fetal and infant growth improves the predictive performance. Prediction Model 1 included the sex of the child, maternal diabetes status, maternal age, pre-pregnancy BMI, smoking status during pregnancy, ethnicity, parity, duration of breastfeeding and offspring birthweight. In Prediction Model 2, we added fetal growth rate at gestational week 34, and in Prediction Model 3, we further added individual infant growth posterior group membership probabilities. We compared model discrimination using the area under the curve (AUC) of the receiver operating characteristic (ROC) curve of the three models using DeLong’s test for two correlated ROC curves.

To improve the models’ performance internally, we used 5-fold cross validation with each model. Using this method, the cohort was split into 5 equal-sized groups and the model was developed on 4/5th of the data (training data) and tested on the remaining 1/5th of the data (testing data). This was done over five iterations with each iteration shifting the training and testing data so that each 1/5th of the data was used as training data four times and as testing data one time.

All analyses were performed using R version 4.3.0 (2023-04-21) (R Foundation for Statistical Computing, Vienna, Austria).

## Results

### Study population

**Fig. 1:**
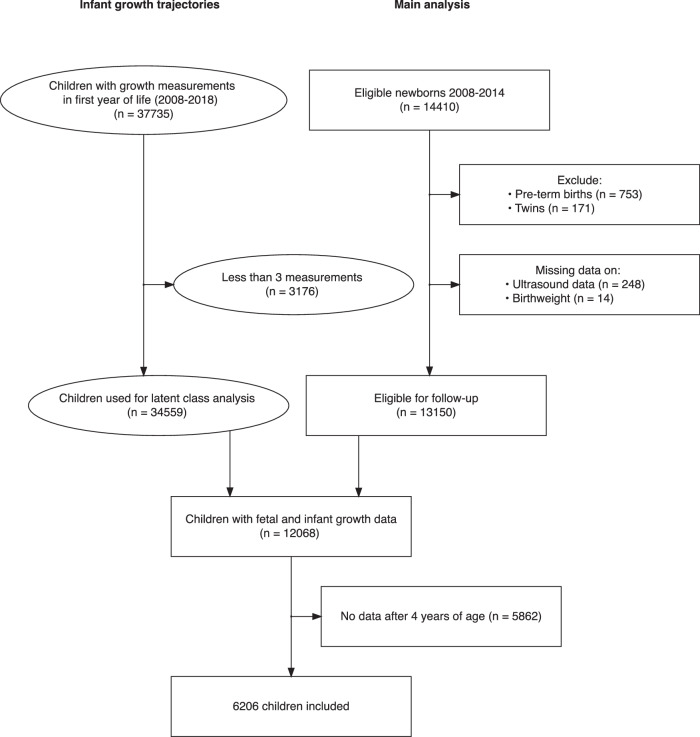
Flowchart of inclusion of participants. Round shapes show the inclusion of children used for latent class trajectory analysis of growth in the first year. Rectangular shapes show inclusion of children in analysis of fetal and infant growth and overweight in the main analysis.

We identified 13,150 newborns born 2008–2014, which were eligible for five-year follow-up. For the infant growth latent class analysis, we identified 37,735 children born 2008 to 2018 who were eligible for one-year follow-up, 34,559 of whom had sufficient data for latent class analysis. There were 12 068 children whose fetal and infant growth data were available. In total, 6206 children with fetal and infant growth data and childhood follow-up data were included in the main analysis (Fig. 1).

Table 1 shows the characteristics of the population. On average, women were 31 (SD: 5) years old, had a BMI of 23.6 kg/m^2^ (SD: 4.4), and 91% were Caucasian. Children had an average birthweight of 3541 g (SD: 466), and 17% were overweight at 5–9 years of age. The majority were 6–8 years old at follow-up; 41% were 6 years old, 43% were 7 years old, and 13% were 8 years old.

**Table 1:** Baseline characteristics.

		Infant growth group		
	Overall	Average	Accelerated	Decelerated
**Characteristic**	*N* = 6210	*N* = 3092	*N* = 468	*N* = 2650
Maternal age (years), Mean (SD)	31 (5)	30 (5)	30 (5)	31 (5)
Pre-pregnancy weight (kg), Mean (SD)	67 (13)	67 (13)	67 (14)	66 (13)
Missing	88	41	7	40
Height (cm), Mean (SD)	168 (7)	168 (7)	168 (7)	168 (7)
Missing	70	38	7	25
Pre-pregnancy BMI (kg/m^2^), Mean (SD)	23.6 (4.4)	23.7 (4.5)	23.9 (4.7)	23.4 (4.3)
Missing	50	28	5	17
Pre-pregnancy BMI group, *n* (%)				
Underweight	256 (4.2)	120 (3.9)	22 (4.8)	114 (4.3)
Normal weight	4191 (68)	2051 (67)	300 (65)	1840 (70)
Overweight	1157 (19)	602 (20)	92 (20)	463 (18)
Obese	556 (9.0)	291 (9.5)	49 (11)	216 (8.2)
Missing	50	28	5	17
Smoking, *n* (%)	363 (5.9)	204 (6.6)	41 (8.8)	118 (4.5)
Missing	33	17	3	13
Ethnicity, *n* (%)				
Caucasian	5615 (91)	2769 (90)	415 (90)	2431 (92)
Other	542 (8.8)	295 (9.6)	46 (10.0)	201 (7.6)
Missing	53	28	7	18
Parity (nullipara), *n* (%)	2432 (46)	1237 (47)	237 (60)	958 (42)
Missing	923	465	73	385
Diabetes, *n* (%)				
No diabetes	5961 (96)	2973 (96)	449 (96)	2539 (96)
Gestational diabetes	225 (3.6)	109 (3.5)	19 (4.1)	97 (3.7)
Pre-gestational diabetes	24 (0.4)	10 (0.3)	0 (0)	14 (0.5)
Cesarean section, *n* (%)	1042 (17)	556 (18)	107 (23)	379 (14)
Missing	56	25	6	25
Fetal growth rate week 34 (g/week), Mean (SD)	204 (28)	201 (28)	190 (28)	210 (28)
Size at birth, *n* (%)				
AGA	5029 (81)	2536 (82)	329 (70)	2164 (82)
SGA	700 (11)	381 (12)	124 (26)	195 (7.4)
LGA	481 (7.7)	175 (5.7)	15 (3.2)	291 (11)
Birthweight (g), Mean (SD)	3541 (466)	3481 (444)	3251 (447)	3662 (460)
Birthweight *Z*-score, Mean (SD)	−0.13 (0.99)	−0.22 (0.95)	−0.63 (0.98)	0.07 (0.98)
AC *Z*-score week 19–25, Mean (SD)	0.36 (0.84)	0.37 (0.84)	0.40 (0.83)	0.35 (0.84)
Missing	195	94	18	83
HC *Z*-score week 19–25, Mean (SD)	−0.24 (0.61)	−0.22 (0.62)	−0.21 (0.62)	−0.27 (0.60)
Missing	195	94	18	83
FL *Z*-score week 19–25, Mean (SD)	−0.33 (0.62)	−0.34 (0.64)	−0.33 (0.63)	−0.32 (0.62)
Missing	195	94	18	83
Sex (boy), *n* (%)	3081 (50)	1539 (50)	265 (57)	1277 (48)
Gestational age (days), Mean (SD)	281 (8)	280 (8)	278 (10)	282 (8)
No. of ultrasound scans, Mean (SD)	1.90 (1.31)	1.92 (1.30)	2.09 (1.58)	1.86 (1.26)
Breastfeeding duration, *n* (%)				
No breastfeeding	788 (15)	419 (16)	89 (24)	280 (13)
0–4 months	1012 (20)	514 (20)	122 (32)	376 (17)
≥ 4 months	3385 (65)	1673 (64)	167 (44)	1545 (70)
Missing	1.025	486	90	449
Overweight age 5–9 years (WHO), *n* (%)	1081 (17)	642 (21)	135 (29)	304 (11)
BMI-for-age *Z*-score at 5–9 years, Mean (SD)	0.12 (1.01)	0.26 (1.00)	0.46 (1.03)	−0.12 (0.97)
Age at BMI measurement (years), Mean (SD)	7.16 (0.70)	7.16 (0.69)	7.22 (0.72)	7.15 (0.70)

Infant growth groups were created using latent class trajectory analysis. *Z*-scores of birthweights calculated using the formula by Marsal et al. [19] and AC, HC, and FL using the formulas by Chitty et al. [21–23].

*AC* abdominal circumference, *HC* head-circumference, *AGA* appropriate for gestational age, *SGA* small for gestational age (birthweight < 10th percentile), *LGA* large for gestational age (birthweight > 90th percentile).

### Fetal growth

In our cohort, 54% had one ultrasound scan performed after week 18 (routine care), 24% had two scans performed, and 22% had more than two. At week 34, the estimated mean fetal weight was 2397 g (95% CI: 2389–2405), and the mean fetal growth rate was 204 g/week (SD: 28) (Supplementary Fig. S2).

Those who underwent additional scans were more likely to have diabetes, had higher caesarean section rates, and had slightly lower birthweights, while their childhood BMI-*z* scores and overweight frequencies were similar (Supplementary Table S1).

### Infant growth

**Fig. 2:**
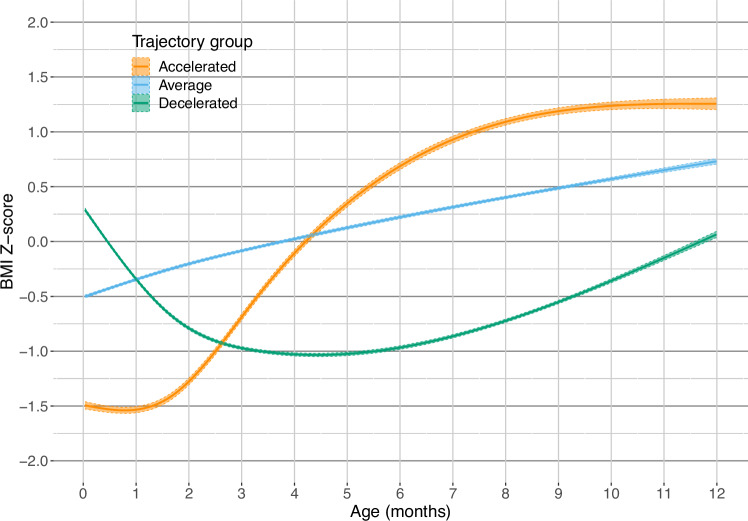
Trajectories of infant growth. Groups are created using latent class analysis. *Z*-scores were created using WHO reference material.

We identified three distinct patterns of growth in the first year of life: average (51%), decelerated (40%), and accelerated growth (9%) (Fig. 2). The mean probabilities of belonging to the assigned group were 79% for the average group, 83% for the decelerated growth group, and 82% for the accelerated group (Supplementary Fig. S3 and Table S2). Group separation measured by relative entropy was 61%.

### Associations with overweight

According to the adjusted model, every 10 g/week increase in the fetal growth rate in week 34 was associated with a 1.09 (adjusted, 95% CI: 1.06–1.12) increase in the odds of being overweight at 5–9 years of age (Table 2). For every 10 g/week in week 28, this was 1.16 (adjusted, 95% CI: 1.11–1.21). Children with decelerated infant growth had a lower risk of being overweight, while children with accelerated infant growth had an increased risk compared to the average group (Table 2). When combining fetal growth at week 34 and infant growth, using children with an average growth rate both in fetal life and infancy as a reference, children with fast fetal growth and subsequent accelerated infant growth had the greatest risk of being overweight at 5–9 years but represented a very small group (*n* = 59). There was also an increased risk among children with average fetal growth and subsequent infant accelerated growth and among children with fast fetal growth and average infant growth but not among those with fast fetal growth and infant decelerated growth (Table 3). There was no additional effect, other than the multiplicative effect, between the fetal and infant growth groups (*p*-value for interaction: 0.54). The combinations of fetal growth at week 28 and infant growth are shown in Supplementary Table S3.

**Table 2:** Associations of fetal growth (per 10 g/week increase) in week 34 and infant growth with overweight in two separate models.

	Unadjusted	Adjusted
Characteristic	OR (95% CI)	OR (95% CI)
**Fetal growth**		
Fetal growth rate (10 g/week)	1.11 (1.08–1.14)	1.09 (1.06–1.12)
**Infant growth**		
Growth class		
Average	Reference	Reference
Accelerated	1.55 (1.24–1.92)	1.52 (1.20–1.90)
Decelerated	0.49 (0.43–0.57)	0.51 (0.44–0.59)

Estimates are odds ratios with 95% confidence intervals. Adjusted model includes maternal pre-pregnancy BMI, age, parity, smoking, and breastfeeding duration.

**Table 3:** Odds ratios (95% CI) of overweight in the different combinations of fetal growth groups in week 34 (slow: ≤184 g/week, average: 185–219 g/week, fast: ≥220 g/week) and infant growth group (average, decelerated, and accelerated).

Infant growth			
Fetal growth	Average	Accelerated	Decelerated
**Unadjusted**			
Average	Reference, *n* = 1504	2.04 (1.49–2.80), *n* = 208	0.43 (0.34–0.53), *n* = 1265
Slow	0.71 (0.56–0.89), *n* = 850	1.06 (0.74–1.53), *n* = 201	0.28 (0.19–0.41), *n* = 502
Fast	1.67 (1.36–2.05), *n* = 734	2.57 (1.50–4.41), *n* = 59	0.82 (0.66–1.02), *n* = 883
**Adjusted**			
Average	Reference, *n* = 1504	1.96 (1.41–2.73), *n* = 208	0.45 (0.35–0.56), *n* = 1265
Slow	0.72 (0.57–0.91), *n* = 850	1.05 (0.72–1.53), *n* = 201	0.30 (0.21–0.44), *n* = 502
Fast	1.52 (1.23–1.88), *n* = 734	2.03 (1.16–3.56), *n* = 59	0.79 (0.63–0.98), *n* = 883

Unadjusted and adjusted for maternal pre-pregnancy BMI, age, parity, smoking, and breastfeeding duration.

*OR* odds ratio, *CI* confidence interval.

At week 34, an increased rate of abdominal growth was associated with being overweight at 5–9 years (OR: 1.50, [95% CI: 1.31–1.72], adjusted OR (aOR): 1.36, [95% CI: 1.18–1.56]) and, to a lesser extent, an increased growth rate of head circumference (OR: 1.34, [95% CI: 1.03–1.75], aOR: 1.30, [95% CI: 0.99–1.71]), while the growth rate of femur length showed no association (OR: 0.66, [95% CI: 0.22–1.99], aOR: 0.45, [95% CI: 0.14–1.40]) (Supplementary Fig. S4).

### Prediction model

**Fig. 3:**
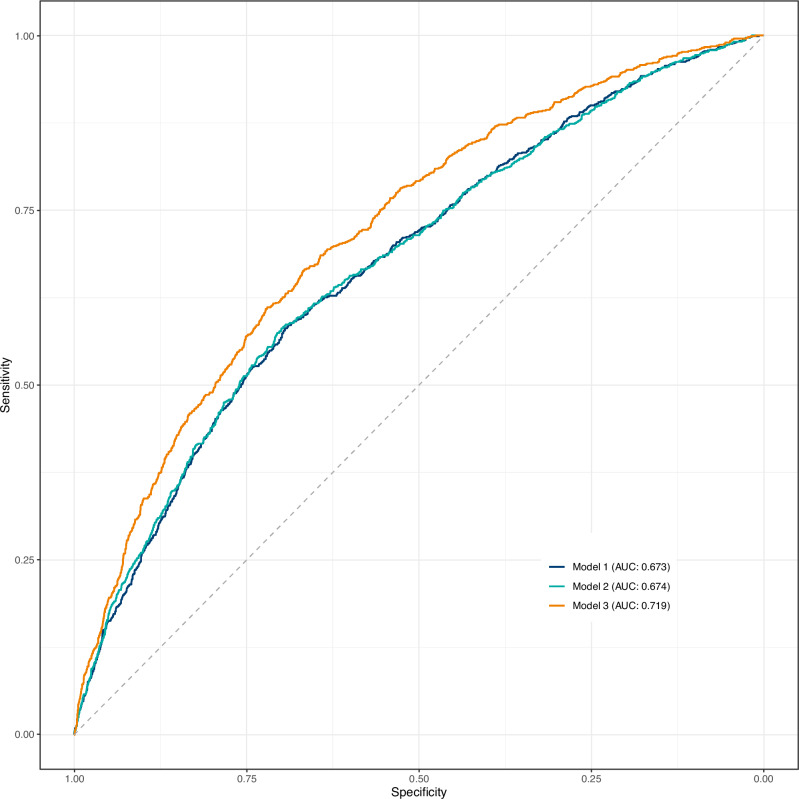
ROC curves of three prediction models. Prediction Model 1 includes sex of the child, maternal diabetes, maternal age, pre-pregnancy BMI, smoking during pregnancy, ethnicity, parity, duration of breastfeeding and birthweight. Prediction Model 2 includes variables in Prediction Model 1 and fetal growth rate in week 34. Prediction Model 3 includes variables in Prediction Model 2 and infant growth group.

According to Prediction Model 1, with neither fetal nor infant growth, the AUCROC was 0.67 (95% CI: 0.65–0.69). When the fetal growth rate at week 34 was added (Prediction Model 2), the AUCROC remained unchanged at 0.67 (95% CI: 0.66–0.69), but when the infant growth group was included (Prediction Model 3), it increased by 0.04 (95% CI: 0.03–0.06, *p* < 0.001) to 0.72 (95% CI: 0.70–0.74) (Fig. 3).

## Discussion

In this longitudinal cohort study, we found that both fetal growth and infant growth were independently associated with being overweight at the age of 5–9 years. When analyzing the combination of fetal and infant growth, we found marked differences in the risk of overweight dependent on the combined patterns of fetal and infant growth. Despite the association between fetal growth and childhood overweight, fetal growth had no predictive value when added to a model already containing birthweight. However, infant growth markedly improved the prediction model, resulting in a prediction model with moderate performance.

### Fetal growth and overweight

An increased fetal growth rate at weeks 28 and 34 was associated with later overweight, similar to studies from the Generation R and Project Viva cohorts, which reported that fetal weight in the second trimester, weight gain from the second to third trimesters and weight gain from the third trimester to birth are associated with overweight at five to six years of age [5, 6]. These studies measured only differences in weight rather than modeling fetal growth, and unlike the current study, most studies on fetal growth and later overweight have used birthweight as a measure of fetal growth [26]. An increase in the growth rate of the abdominal circumference showed a slightly stronger association with being overweight than did the growth rate of the head, while there was no association with femur growth. Greater fetal abdominal circumference is associated with greater fat mass in neonates [27] and young children [28]. We therefore, speculate that the body composition of higher fat and lower lean mass in the fetus might account for the increased risk of overweight we observed with an increased fetal growth rate. However, the confidence intervals were wide, and it should be noted that this analysis was performed on those who underwent ultrasound examinations in addition to routine care and who might not be representative of the general population.

### Infant growth and overweight

We found a decreased risk of being overweight in infants with decelerated growth and an increased risk in the accelerated growth group compared to children in the average group. This association is probably partially explained by the accelerated group having a lower fetal growth rate as the birthweight *z*-score was lower and the prevalence of small for gestational age birthweight was greater in this group, and children with fetal growth restriction are known to be at increased risk of overweight if they are subjected to excessive accelerated growth [29]. It should be noted that children with accelerated or decelerated growth can be born with a BMI *Z*-score of more than 0 or less than 0, respectively, and can be assigned to the group with a matching shape. Similar results of a low trajectory being protective and high or accelerating trajectories being associated with increased risk of overweight have also been reported by others [5, 9, 10, 30, 31].

### Interaction between fetal and infant growth

When investigating the relationship between fetal and infant growth, we found that fast fetal growth was positively associated with overweight, but not if followed by decelerated growth; average fetal growth was associated with overweight, but only if followed by accelerated growth; and slow fetal growth was not associated with overweight, regardless of infant growth. Generally, it seems that infant growth has a strong impact on the risk of becoming overweight later in life, but the risk is influenced by fetal growth. Children with fast fetal growth will often be born with a higher birthweight and few will continue with accelerated infant growth (*n* = 59), making interpretations of this group difficult. Additionally, infants with accelerated growth had the smallest birthweight, indicating that accelerated growth is often observed in those born small. Children with slow fetal growth and accelerated growth in infancy did not have an increased risk of overweight, contrary to what we might expect from the literature on catch-up growth in children born small-for-gestational age who are at increased risk [29]. We probably did not find evidence for this association because these children were not necessarily growth restricted, and appropriate catch-up growth in infancy is probably beneficial if born small [32, 33], whereas children with average or fast fetal growth and subsequent accelerated growth had an increased risk of overweight. There were some differences in the characteristics of the children in the three infant growth groups, i.e., a higher rate of maternal smoking, nulliparity, no breastfeeding, and cesarean section in the accelerated growth group. Except from delivery mode, these variables were included a priori in the adjusted logistic regressions. Many studies have investigated either fetal or infant growth in relation to the later development of overweight, but few have investigated the interaction, even though fetal growth is immediately followed by infant growth. Accelerated fetal growth followed by accelerated infant growth had the largest association with BMI at six years in the Generation R study, while accelerated fetal growth followed by decelerated or normal infant growth showed small or no associations with high BMI [5], similar to our results. The same tendencies were observed for fat mass index suggesting that the BMI is increased due to increased fat mass rather than lean mass [5].

### Prediction of childhood overweight

Although we found an association between fetal growth rate and overweight, adding fetal growth rate to a prediction model already containing known risk factors, including birthweight, did not improve the predictive performance, suggesting that birthweight is as good as fetal growth rate in predicting overweight. One possible explanation for why fetal growth did not improve the prediction is that birthweight was also used in creating the fetal growth trajectories, making the trajectories and birthweight highly correlated. Nevertheless, we believe that the fetal growth rate holds importance, as compared to birthweight, simply because it precedes birthweight, making earlier detection possible. Even if the possibilities for interventions for altered fetal growth are sparse, in cases of diabetes during pregnancy, excessive gestational weight gain, smoking, or hypertension, there are possibilities to improve fetal growth. Unlike fetal growth, the infant growth trajectory was both associated with overweight and improved the performance of the prediction model. Additionally, infant growth was captured more frequently and with greater precision than fetal growth. In an individual-level systematic review, Druet et al reported a similar performance of a prediction model with birthweight SD-score, sex, and maternal BMI, with an AUCROC of 0.68, which increased even more than in our models, to 0.77 when including infant growth [10]. However, discrimination remains moderate for clinical use.

### Strengths and limitations

In Denmark, health care is free for everyone, and combined with frequent visits from health nurses during infancy, it provides detailed growth data on a large population representing all parts of society. Furthermore, the cohort consists of recent data, and with the rapid changes in demographics, especially BMI, in recent decades, older cohorts might not be comparable to today’s populations. By using latent class analysis, we define the groups by the patterns in growth that might not be apparent beforehand.

This study is not without limitations. The use of routinely collected ultrasound scans is limited by the number of scans offered in routine antenatal care, which is two in Denmark. To maximize the number of scans included in the analysis, we included additional scans performed on any indication, which might introduce bias since those offered additional scans could have underlying causes for these scans. However, additional scans are not exceptional; almost half of the population had additional scans performed, and those who did were largely similar to those receiving routine care, except for a higher prevalence of small for gestational age birthweight and cesarean section. Therefore, we believe that the impact of bias introduced is minimal.

By using routinely collected data, we are limited by the information available. As such, residual confounding may persist. For example, information on gestational weight gain was not available, which could be a confounder.

We estimated weight status over a relatively wide age range. This was done to include as many children as possible, as stricter criteria could have increased the risk of selection bias. Additionally, overweight at older ages is increasingly associated with a risk of sustained obesity [1]. There are several limitations to the use of BMI as a measure of overweight and obesity. Body composition changes during childhood, and for individuals with the same BMI, there are different body fat percentages in different populations [34]. Additionally, there are large inter- and intra-individual differences in the relationship between BMI and subsequent health outcomes. However, BMI remains a valid routinely measured estimate of body fat [34].

In the latent class, uncertainty around group membership probabilities is not considered after an individual is assigned to a group.

Further studies using frequent, serial fetal weight estimates, and including infant growth measures are needed to better understand the importance of fetal and infant growth patterns and their interaction in future weight and metabolic health.

## Conclusion

In conclusion, fetal and infant growth were independently associated with being overweight at age 5–9 years of age. However, distinct combinations of fetal and infant growth showed marked differences in risk, suggesting that growth in each period influences each other in relation to later weight status. Even if fetal and infant growth were both associated with overweight, the fetal growth rate provided no additional predictive capabilities for childhood overweight over a prediction model with known risk factors, including birthweight. However, infant growth improved the predictive power. In the future, to prevent the development of childhood overweight, infant growth patterns could be used as an early warning sign but with size at birth in mind.

## Supplementary information

Supplemental material

## Data Availability

The data that support the findings of this study are available from Central Denmark Region but restrictions apply to the availability of these data, which were used under license for the current study, and so are not publicly available. Data are however available from the authors upon reasonable request and with permission of Central Denmark Region and the Danish Data Protection Agency.
