# Supplementary material for: The association of fetal growth rate and growth in first year of life with childhood overweight: a cohort study
Source: Int J Obes (Lond). 2024 Sep 21;48(12):1822–30. doi: 10.1038/s41366-024-01637-w (PMC11584398; doi:10.1038/s41366-024-01637-w)
Supplement: Supplementary file 1 — Supplemental material [file 41366_2024_1637_MOESM1_ESM.pdf]

# The association of fetal growth rate and growth in first year of life with childhood overweight: A cohort study Supplementary

**Authors:**

Magnus Leth-Møller[1, 2, 3], magnusmoeller@clin.au.dk

Ulla Kampmann[1,3]

Susanne Hede[4]

Per G. Ovesen[1,2,3]

Adam Hulman*[3,5]

Sine Knorr*[1,3]

*Adam Hulman and Sine Knorr contributed equally.

Magnus Leth-Møller is the corresponding author.

Affiliations:

[1] Dept. of Clinical Medicine, Aarhus University, Palle Juul-Jensens Boulevard 99, 8200 Aarhus N, Denmark

[2] Dept. of Obstetrics and Gynaecology, Aarhus University Hospital, Palle Juul-Jensens Boulevard 99, 8200 Aarhus N, Denmark

[3] Steno Diabetes Center Aarhus, Aarhus University Hospital, Palle Juul-Jensens Boulevard 11, 8200 Aarhus N, Denmark

[4] Healthcare service for families, Aarhus Municipality, Grøndalsvej 2, 8260 Viby J, Denmark

[5] Dept. of Public Health, Aarhus University, Bartholins Allé 2, 8000 Aarhus C, Denmark

# Directed acyclic graph

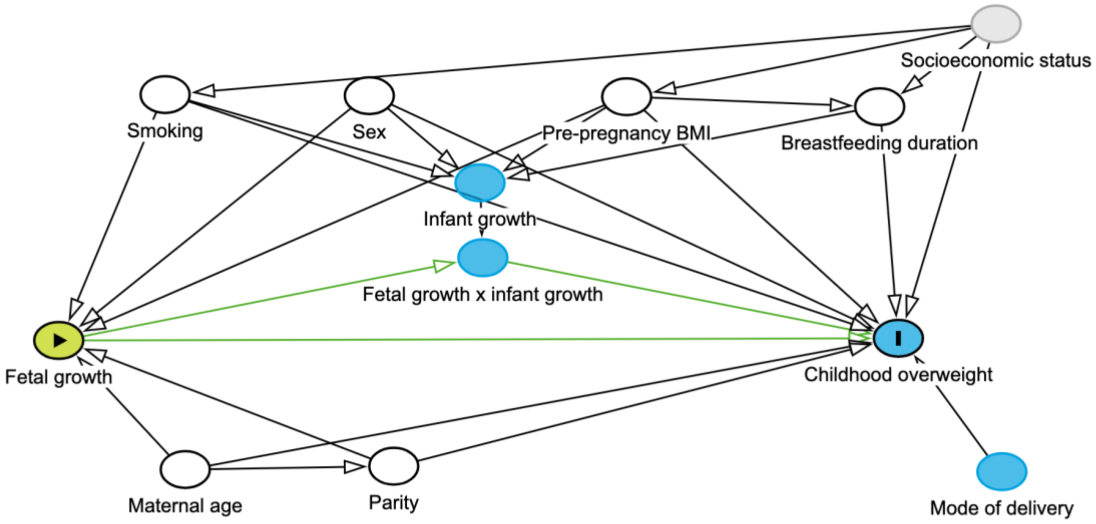


*Figure S1: Directed acyclic graph of the association between fetal and infant growth and later overweight.*

## Characteristics based on ultrasound scans

| | No. ultrasound scans | |
|---|---|---|
| **Characteristic** | **Extra**, N = 2,882 | **Routine**, N = 3,324 |
| Maternal age (years), Mean (SD) | 31 (5) | 30 (5) |
| Pre-pregnancy weight (kg), Mean (SD) | 68 (15) | 66 (12) |
| Height (cm), Mean (SD) | 168 (7) | 168 (6) |
| BMI (kg/m$^2$), Mean (SD) | 23.9 (4.8) | 23.3 (4.1) |
| Smoking, n (%) | 176 (6.1) | 187 (5.7) |
| Ethnicity, n (%) | | |
| Caucasian | 2,576 (90) | 3,036 (92) |
| Other | 276 (9.7) | 263 (8.0) |
| Parity (nullipara), n (%) | 1,052 (42) | 1,379 (49) |
| Diabetes, n (%) | | |
| No diabetes | 2,647 (92) | 3,310 (100) |
| GDM | 211 (7.3) | 14 (0.4) |
| T2DM | 12 (0.4) | 0 (0) |
| T1DM | 12 (0.4) | 0 (0) |
| Maternal education, n (%) | | |
| No education | 16 (2.3) | 7 (0.8) |
| Primary school | 131 (19) | 133 (15) |
| High school | 130 (19) | 199 (23) |
| Higher education | 405 (59) | 529 (61) |
| Cesarean section, n (%) | 587 (21) | 455 (14) |
| Fetal growth rate in week 34 (g/week), Mean (SD) | 203 (31) | 205 (26) |
| Birthweight (g), Mean (SD) | 3,503 (493) | 3,575 (439) |
| Birthweight Z-score, Mean (SD) | -0.19 (1.06) | -0.08 (0.92) |
| Size at birth, n (%) | | |
| AGA | 2,233 (77) | 2,793 (84) |
| SGA | 410 (14) | 289 (8.7) |
| LGA | 239 (8.3) | 242 (7.3) |
| AC Z-score week 19-25, Mean (SD) | 0.34 (0.86) | 0.39 (0.82) |
| HC Z-score week 19-25, Mean (SD) | -0.27 (0.61) | -0.21 (0.61) |
| FL Z-score week 19-25, Mean (SD) | -0.35 (0.63) | -0.31 (0.61) |
| Sex (boy), n (%) | 1,411 (49) | 1,669 (50) |
| Gestational age (days), Mean (SD) | 281 (9) | 281 (8) |
| No. of ultrasound scans, Mean (SD) | 2.93 (1.27) | 1.00 (0.00) |
| Breastfeeding duration, n (%) | | |
| No breastfeeding | 402 (16) | 386 (14) |
| 0-4 months | 498 (20) | 513 (19) |
| ≥ 4 months | 1,537 (63) | 1,846 (67) |
| Overweight in childhood, n (%) | 524 (18) | 556 (17) |
| BMI-for-age Z-score at 4-9 years, Mean (SD) | 0.14 (1.02) | 0.09 (1.00) |
| Age at BMI measurement (years), Mean (SD) | 7.11 (0.71) | 7.20 (0.68) |

Table S1. Characteristics of those receiving routine ultrasound care and those receiving additional scans. Z-scores of birthweight calculated using Marsal[1]. AGA: Appropriate for gestational age, SGA: Small for gestational age (<10$^{th}$ percentile), LGA: Large for gestational age (>90$^{th}$ percentile).

## Fetal growth trajectory

Cubic mixed model of fetal growth.

```
df$ga2 <- ga^2
df$ga3 <- ga^3

trajectory <- nlme::lme(efw~ga+ga2+ga3,
                  data=df,
                  random=~ga+ga2|child_id,
                  na.action=na.exclude,
                  control = lmeControl(opt='optim'))
```

To estimate trajectories of abdominal circumference, head circumference, and femur length, we created mixed model regressions. Abdominal circumference and head circumference at birth was used in continuation of ultrasound estimates, if available. A minimum of two measurements, either using ultrasound examination or at birth, were required. The data was restricted to those having two measures of all three variables. See below for model specifications.

```r
# ga = gestational age
data$ga2 <- data$ga^2
data$ga3 <- data$ga^3

# Abdominal circumference
trajectory_abdominal_circumference <-
        lme(abdominal_circumference~ga+ga2+ga3,
                      data=data,
                      random=~ga|id,
                      na.action=na.exclude,
                      control = lmeControl(opt='optim'))

# Head circumference
trajectory_head_circumference <-
        lme(head_circumference~ga+ga2+ga3,
                      data=data,
                      random=~ga|id,
                      na.action=na.exclude,
                      control = lmeControl(opt='optim'))

# Femur length
trajectory_femur_length <-
        lme(femur_length~ga+ga2+ga3*weight_status,
                      data=data,
                      random=~ga|id,
                      na.action=na.exclude,
                      control = lmeControl(opt='optim'))
```

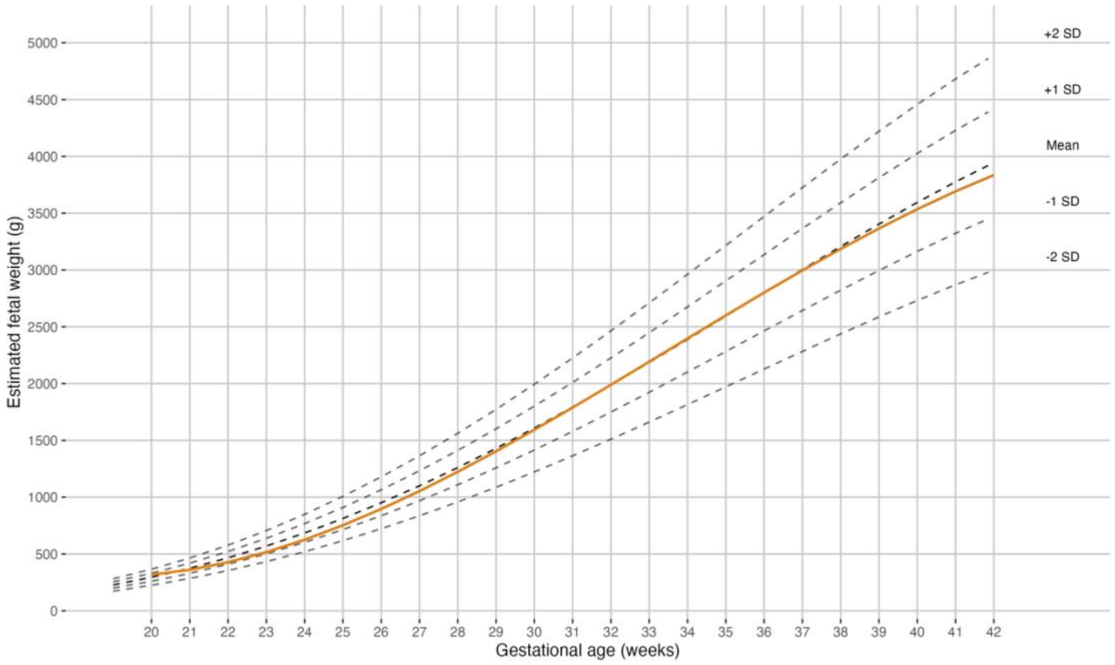


*Figure S2: Fetal growth trajectory. Dashed lines shows mean +/- 2SD using a Scandinavian reference material*[19]

## Infant growth

Latent class growth mixture model of infant growth using mixed model cubic splines. We optimized the model using a grid search with 20 repetitions with a maximum of 100 iterations each. See below.

```
# 1 group to initiate
lcgmm_spline_1 <-
        lcmm::hlme(zbmi ~ Epi::Ns(age, knots = c(0.3, 50, 100, 363)),
             subject = "id",
             random = ~1,
             ng = 1,
             data = Data)

# Gridsearch solution - 3 classes
lcgmm_spline_3_z_grid <-
        lcmm::gridsearch(rep = 20, maxiter = 100, minit = lcgmm_spline_1,
                hlme(zbmi ~ Epi::Ns(age, knots = c(0.3, 50, 100, 363))
,
                     subject = "id",
                     random = ~1,
                     mixture =  ~ Ns(age, knots = c(0.3, 50, 100, 363)
),
                     ng = 3,
                     data = Data))
```

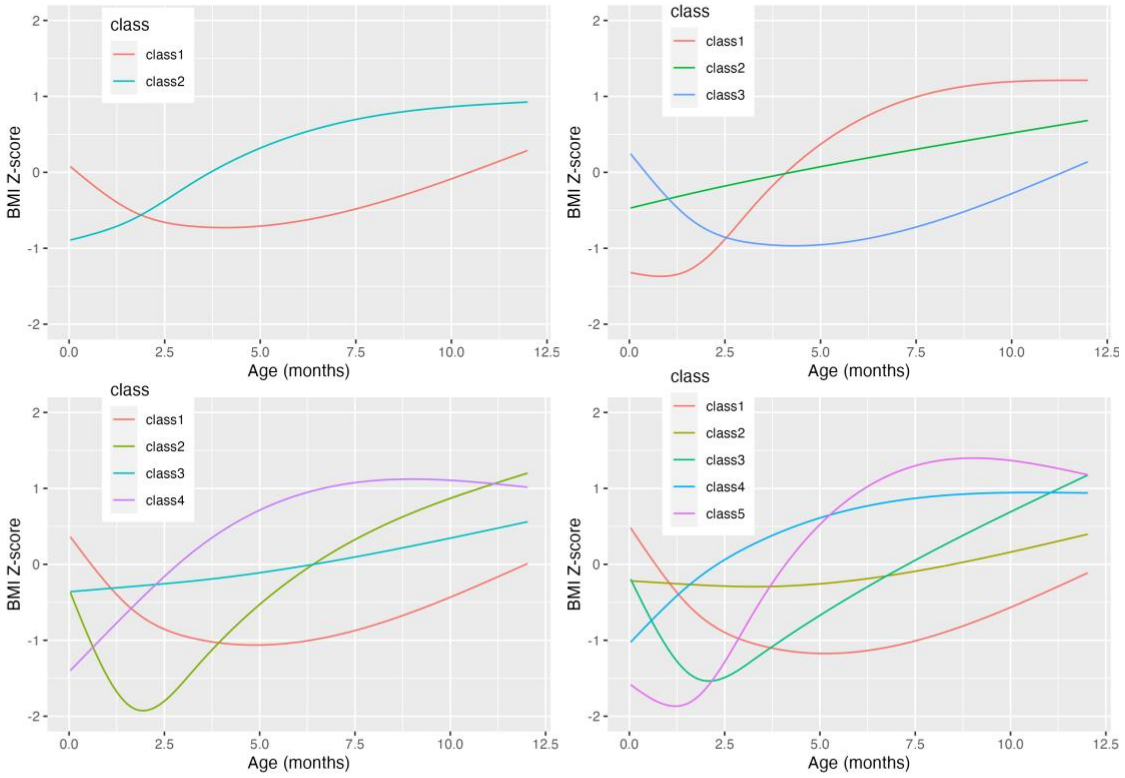


*Figure S3. Trajectories of the 2-5 group solutions of BMI-z trajectories in infancy.*

**Posterior probabilities**

| No. of groups | Group | Proportion (%) | Mean posterior probability of assignment (%) | | | | | Relative entropy (%) | BIC | AIC |
|---|---|---|---|---|---|---|---|---|---|---|
| | | | 1 | 2 | 3 | 4 | 5 | | | |
| 2 | 1 | 63 | 89 | 11 | | | | 61 | 461128.18 | 461034.85 |
| | 2 | 37 | 14 | 86 | | | | | | |
| 3 | 1 | 9 | 82 | 17 | 1 | | | 61 | 434161.1 | 434025.9 |
| | 2 | 51 | 7 | 79 | 14 | | | | | |
| | 3 | 40 | 0.4 | 16 | 84 | | | | | |
| 4 | 1 | 27 | 81 | 2 | 17 | 0 | | 65 | 450175.89 | 449997.7 |
| | 2 | 6 | 5 | 77 | 13 | 6 | | | | |
| | 3 | 55 | 12 | 3 | 79 | 6 | | | | |
| | 4 | 12 | 0 | 4 | 15 | 81 | | | | |
| 5 | 1 | 19 | 78 | 17 | 4 | 0 | 0 | 63 | 427076.12 | 426856.41 |
| | 2 | 51 | 10 | 75 | 7 | 8 | 0 | | | |
| | 3 | 10 | 5 | 15 | 71 | 6 | 3 | | | |
| | 4 | 18 | 0 | 15 | 4 | 77 | 4 | | | |
| | 5 | 3 | 0 | 1 | 7 | 13 | 79 | | | |

Table S2. Posterior probabilities, relative entropy, Bayesian information criterion (BIC) and Akaike information criterion (AIC) for 2-5 group solutions.

## Fetal growth in week 28 and infant growth and later overweight

| | Infant growth | | |
|---|---|---|---|
| **Fetal growth** | Average | Accelerated | Decelerated |
| **Unadjusted** | | | |
| Average | Reference, n = 1738 | 1.79 (1.34-2.4), n =240 | 0.44 (0.36-0.54), n =1549 |
| Slow | 0.64 (0.52-0.8), n =877 | 0.97 (0.67-1.39), n =194 | 0.28 (0.2-0.41), n =507 |
| Fast | 1.63 (1.3-2.04), n =473 | 3.72 (1.88-7.36), n =34 | 0.78 (0.61-0.99), n =594 |
| **Adjusted** | | | |
| Average | Reference, n = 1738 | 1.69 (1.24-2.3), n =240 | 0.46 (0.37-0.56), n =1549 |
| Slow | 0.67 (0.53-0.84), n =877 | 0.99 (0.68-1.45), n =194 | 0.31 (0.22-0.45), n =507 |
| Fast | 1.44 (1.14-1.83), n =473 | 2.85 (1.4-5.81), n =34 | 0.74 (0.58-0.95), n =594 |

Table S3. Table 3**.** Odds ratios (95% CI) of overweight in the different combinations of fetal growth groups in week 28 (slow: ≤164 g/week, average: 165-189 g/week, fast: ≥190 g/week) and infant growth group (average, decelerated, and accelerated). Unadjusted and adjusted for maternal pre-pregnancy BMI, age, parity, smoking, and breastfeeding duration. OR: Odds ratio, CI: Confidence interval.

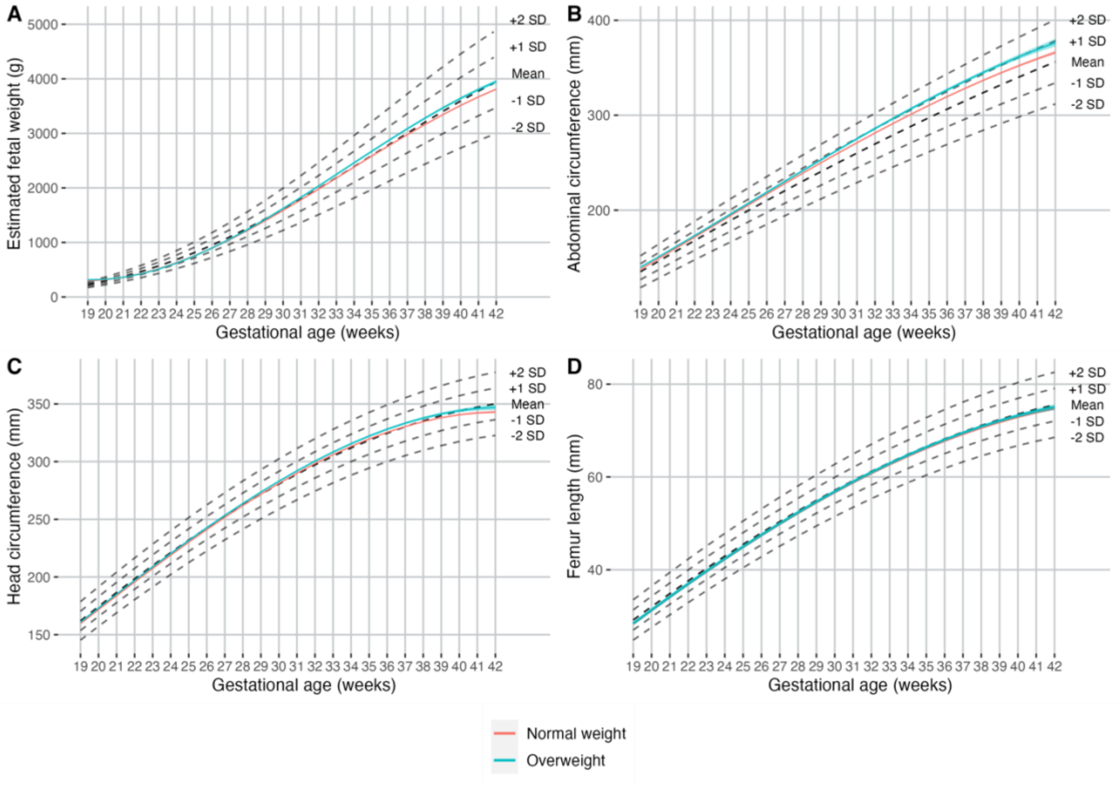


*Figure S4: Trajectories of A: Estimated fetal weight, B: Abdominal circumference, C: Head circumference, and D: Femur length from 19 to 42 weeks of gestation in children with overweight (orange line) and normal weight (blue line) at 5-9 years of age. The colored ribbons around the lines show 95% confidence interval for the model derived estimates. The shaded area shows the 2.5th to 97.5th centile range of data. Dashed lines show mean +/- 2 SD using the formula by Marsal[1] et al for estimated fetal weight and the formulas by Chitty et al for abdominal[2] and head[3] circumference and femur length[4].*

## Supplementary references


1. Marsál, K. *et al*. Intrauterine growth curves based on ultrasonically estimated foetal weights. *Acta Paediatr* **85**, 843–8 (1996).
2. Chitty, L. S., Altman, D. G., Henderson, A. & Campbell, S. Charts of fetal size: 3. Abdominal measurements. *BJOG: An International Journal of Obstetrics & Gynaecology* **101**, 125–131 (1994).
3. Chitty, L. S., Altman, D. G., Henderson, A. & Campbell, S. Charts of fetal size: 2. Head measurements*. *BJOG: An International Journal of Obstetrics & Gynaecology* **101**, 35–43 (1994).
4. Chitty, L. S., Altman, D. G., Henderson, A. & Campbell, S. Charts of fetal size: 4. Femur length. *BJOG: An International Journal of Obstetrics & Gynaecology* **101**, 132–135 (1994).
